# Inequitable exposure to tobacco product litter among adolescents in California, USA

**DOI:** 10.18332/tpc/200195

**Published:** 2025-02-10

**Authors:** Benjamin W. Chaffee, Omara Farooq, Elizabeth T. Couch, Candice D. Donaldson, Nancy F. Cheng, Stuart A. Gansky, Xueying Zhang

**Affiliations:** 1 Center for Tobacco Control Research and Education, University of California San Francisco, San Francisco, United States; 2 Division of Oral Epidemiology and Dental Public Health, University of California San Francisco, San Francisco, United States; 3 California Tobacco Prevention Program, California Tobacco Control Branch, California Department of Public Health, Sacramento, United States

**Keywords:** tobacco products, adolescent health, health inequities, environment and public health

## Abstract

**INTRODUCTION:**

Tobacco waste is a costly, widespread blight and environmental toxicant that is not distributed equally across geographical areas. This investigation reports on the prevalence of noticing tobacco litter and potential inequities in tobacco litter exposure among adolescents in California, USA.

**METHODS:**

Data from the 2023 Teens, Nicotine, and Tobacco (TNT) Online Survey (N=4910), a statewide, online cross-sectional survey of California adolescents aged 12–17 years, were analyzed for the self-reported frequency of noticing tobacco product litter. All participants were asked to report how often they notice tobacco product litter (closed-ended response options: almost always, sometimes, once in a while, never). Survey-weighted multivariable regression models were fitted to quantify the odds of noticing tobacco litter ‘almost always’ according to participant characteristics (age, sex, gender/sexual identity, race/ethnicity, location, family finances, and own and household tobacco use). Data were weighted for geographical and demographic representativeness and response quality.

**RESULTS:**

The prevalence of noticing tobacco litter almost always was 44.6% overall and higher among participants who identified as Hispanic/Latino (50.9%) or LGBTQ+ (53.3%), lived in a small town (57.8%), or whose families were financially disadvantaged (52.7%). These inequities persisted in multivariable models, including adjustment for own and household tobacco use. For example, Hispanic/Latino participants had 1.66-times the adjusted odds of almost always noticing tobacco litter (95% CI: 1.32–2.07; reference: non-Hispanic White); the adjusted odds ratio for LGBTQ+ identity was 1.39 (95% CI: 1.04–1.87; reference: non-LGBTQ+).

**CONCLUSIONS:**

The pervasive exposure to tobacco litter observed in this study suggests a need for stronger efforts to reduce tobacco waste, with an emphasis on advancing equity.

## INTRODUCTION

Tobacco waste, cigarette butts in particular, is among the most common forms of waste globally, comprising a source of both blight and toxic chemicals that persist in the environment, threatening living organisms and ecosystems^1-3^. By one estimate, the total cost of tobacco waste to each major US city reaches tens of millions of dollars annually, including costs such as mechanical removal, environmental damage, and fires caused by discarded cigarette butts^4^. As the tobacco product marketplace has diversified, so too have the types of waste in the environment, including e-cigarettes^5^, which may pose additional hazards, for example, from spent batteries or hazardous e-liquid chemicals^6^.

Tobacco waste is not distributed equally across various geographical areas, raising environmental and social justice concerns^7^. A recent simulation model estimated that the density of cigarette butt litter was highest among US census tracts marked by the greatest social vulnerabilities^7^. To our knowledge, little available information examines how self-reported exposure to tobacco product litter varies by social identity and socioeconomic circumstances. The objectives of this investigation were to report on the prevalence of noticing tobacco litter and potential inequities in tobacco litter exposure among a statewide online sample of adolescents in California. As context for this investigation, California is a populous, demographically diverse state with a lengthy history of strong tobacco control measures^8^.

## METHODS

### Study design

The TNT Online Survey is a cross-sectional, internet-based survey of adolescents (eligible: ages 12–17 years and residing in California, US) to inform the activities of the California Tobacco Prevention Program, as conducted by the authors and detailed elsewhere^9,10^. The 2023 TNT Online Survey was administered in English or Spanish to a non-probability sample drawn from multiple online commercial panel vendors (n=5015). Sampling occurred in two independent cycles (May and November 2023); cycles were pooled for analysis. The University of California San Francisco Institutional Review Board approved all study procedures.

### Survey measures

All participants were asked: ‘How often do you notice tobacco product litter (such as cigarette butts, cigar wrappers, or vape packaging) in public spaces such as sidewalks, streets, parks, and beaches?’ (response options: almost always, sometimes, once in a while, never). This item was tested in cognitive interviews with 12 adolescents prior to implementation. Based on cognitive testing, a labeled image of a cigarette butt accompanied the question to improve comprehension. This analysis was limited to the n=4910 participants who responded to this survey item. Analyses that follow considered a response of ‘almost always’ as the primary outcome.

Other survey measures used in this analysis included standard closed-ended items for age, sex assigned at birth, ethnicity, and race. Participants were also asked about their gender identity and sexual orientation, location where they reside, their family financial situation^11^, and whether anyone who now lives with them uses tobacco products (selected from a list). Participants were asked about their own use of tobacco (ever and in the past 30 days) separately for 11 types of products, which were combined for analysis.

### Statistical analysis

Descriptive percentages were calculated using the svy command suite in Stata 16. The main goal of the TNT Online Survey is to provide stable estimates of tobacco product use, and the total sample size was designed accordingly. The statistical power of the present analysis, which depended on the frequency of noticing tobacco litter reported by participants, was not estimated a priori. Weights developed from the 2021 American Community Survey 5-Year Public Use Microdata Sample files increased generalizability to the geographical, gender, and race/ethnicity distribution of California adolescents aged 12–17 years. Additional data quality weights down-weighted the contributions of potentially lower quality responses, as described elsewhere^9,10^. A survey-weighted logistic regression model was fitted for the dichotomized outcome variable noticing tobacco product litter ‘almost always’. Participant characteristics were added as covariables (i.e. age, sex, gender/sexual identity, race/ethnicity, location, family finances, and own and household tobacco use) using listwise deletion for missing observations (n=4682 observations retained). While unadjusted percentages were estimated to describe observed inequities in exposure to tobacco litter, the regression model was fitted to adjust for confounding by participants’ own use (or risk of using) tobacco and tobacco use by someone in their homes. Adjusted odds ratios (AORs) and 95% confidence intervals (CIs) were calculated. All statistical tests were two-tailed and considered statistically significant at p<0.05.

## RESULTS

Exposure to tobacco litter was a pervasive experience among study participants. Overall, 86.4% of survey respondents reported noticing tobacco product litter either almost always (44.6%) or sometimes (41.8%). Fewer participants reported noticing tobacco product litter only once in a while (10.3%) or never (3.3%). How often participants reported noticing litter differed according to participant characteristics (Table 1). Noticing tobacco product litter almost always was highest among LGBTQ+ (53.3%) or Hispanic/Latino (50.9%) participants, among those living in a small town (57.8%) or rural area (53.7%), and among those whose family was financially disadvantaged (52.7%) (Table 1). Participants were also more likely to report noticing tobacco product litter almost always if they used tobacco themselves or lived with someone else who uses tobacco (Table 1).

**Table 1:** Participant characteristics and frequency of noticing tobacco product litter, cross-sectional online survey of adolescents, California, USA, 2023 (N=4910)

*Characteristics*		*Prevalence of noticing tobacco litter almost always^a^*			
	*Weighted %^b^*	*Weighted %^c^*	*p^d^*	*AOR (95% CI)^e^*	*p^f^*
**All**	100	44.6			
**Age** (years)			0.93		
12–13 ®	35.1	44.0		1	
14–15	31.8	45.2		1.05 (0.81–1.35)	0.73
16–17	33.1	44.5		0.96 (0.74–1.24)	0.74
**Sex**			0.95		
Male ®	49.9	44.5		1	
Female	50.1	44.6		0.95 (0.77–1.18)	0.63
**Gender/sexual identity^g^**			0.004		
Not LGBTQ+ ®	87.0	43.3		1	
LGBTQ+	13.0	53.3		1.39 (1.04–1.87)	0.03
**Race/ethnicity**			<0.001		
Non-Hispanic White ®	20.7	38.8		1	
Non-Hispanic Black	4.1	44.0		1.26 (0.83–1.89)	0.27
Hispanic/Latino	53.7	50.9		1.66 (1.32–2.07)	<0.001
Non-Hispanic Asian	14.0	29.4		0.78 (0.55–1.11)	0.17
Other^h^	7.5	46.0		1.41 (0.97–2.05)	0.07
**Location^i^**			<0.001		
City ®	42.3	47.1		1	
Suburb	41.9	37.5		0.74 (0.59–0.93)	0.01
Small Town	10.1	57.8		1.44 (1.01–2.03)	0.04
Rural	5.8	53.7		1.24 (0.73–2.10)	0.42
**Family finances**			<0.001		
Lives comfortably/meets needs ®	73.0	41.6		1	
Just meets/does not meet basics	24.4	52.7		1.35 (1.04–1.74)	0.02
Prefer not to say	2.6	51.6		1.25 (0.70–2.25)	0.45
**People living with you**			<0.001		
No one uses any tobacco^j^ ®	66.6	40.6		1	
Someone uses tobacco^j^	33.4	52.4		1.36 (1.08–1.70)	0.01
**Own tobacco^k^ use**			<0.001		
Never ®	70.8	41.5		1	
Ever, 0 days in past 30 days	17.0	51.2		1.26 (0.93–1.69)	0.13
≥1 day in past 30 days	12.2	53.1		1.27 (0.95–1.70)	0.11

AOR: adjusted odds ratio. ® Reference categories. LGBTQ+: lesbian, gay, bisexual, transgender, questioning, or queer.

a Percentages based on up to N=4910 participants who responded to the tobacco litter question; number may be smaller for some variables due to missing data (N=4682 with no missing values included in regression model).

b Weighted column percent for sample characteristics (may not sum to 100% due to rounding).

c Weighted row percent for noticing tobacco litter (may not sum to 100% due to rounding).

d Weighted chi-squared test (global test over all category levels).

e Weighted logistic regression model adjusted for all covariables shown in the table and limited to N=4682 participants with no missing values for any of the covariables.

f Pair-wise comparison to reference category.

g LGBTQ+ identity was categorized as any participant who indicated that their gender was transgender, ‘I’m not sure yet’, or ‘something else’, who indicated that their sexual orientation was gay or lesbian, bisexual, ‘I’m not sure yet’, or ‘something else’, or individuals who reported a gender that differed from their sex at birth.

h Includes participants who indicated their race was American Indian/Alaska Native, Native Hawaiian/Other Pacific Islander, Other, or selected more than one response option.

i Participants were asked, ‘How would you describe the place where you live?’.

j Includes cigarettes, e-cigarettes, cigars, hookah, and/or smokeless tobacco.

k Includes cigarettes, e-cigarettes, cigars, hookah, smokeless tobacco, nicotine pouches, nicotine tablets lozenges or gummies, and/or heated tobacco.

Participant characteristics continued to be independently associated with the self-reported frequency of noticing tobacco product litter after statistical adjustment for own tobacco use and living with someone who uses tobacco (Table 1). The adjusted odds of noticing tobacco product litter almost always were higher among participants who identified as LGBTQ+ compared to those who did not (AOR=1.39; 95% CI: 1.04–1.87) and among those who identified as Hispanic/ Latino compared to those who identified as non-Hispanic White (AOR=1.66; 95% CI: 1.32–2.07) (Table 1). Compared to respondents who resided in a city, the odds of noticing tobacco litter almost always were higher among those residing in a small town (AOR=1.44; 95% CI: 1.01–2.03) and lower among those residing in a suburb (AOR=0.74; 95% CI: 0.59–0.93) (Table 1). Worse family financial situation was also associated with greater odds of noticing tobacco litter almost always (AOR=1.35; 95% CI: 1.04–1.74) (Table 1).

## DISCUSSION

In this study, more than 80% of adolescents in California reported noticing tobacco litter ‘almost always’ or ‘sometimes’. This experience not only potentially exposes young people to harmful chemicals but may also contribute to perceptions that help normalize tobacco use and normalize the discarding of tobacco waste into the environment. Moreover, inequities were evident, with tobacco litter exposure being reported more frequently among historically marginalized groups, even after accounting for participants’ own tobacco use and use in their household. These findings underscore a need for strong tobacco control and prevention initiatives that emphasize the protection of youth and priority populations to advance health equity.

The survey-based findings of the present study partly align with a recent model that predicted the most exposure to cigarette litter in urban areas and within disadvantaged communities^7^. Earlier work reported that cigarette butts were more likely to be found close to businesses where cigarettes are sold and consumed^12^, and the density of tobacco retailers is greater within neighborhoods with larger racial/ethnic minority populations^7,13^. Also, groups that reported more tobacco litter exposure in this study, notably LGBTQ+ individuals, have been long targeted in pro-tobacco marketing^13^. Reducing tobacco waste may have both direct benefits to individuals (e.g. less exposure to harmful chemicals, less normalization of dangerous behaviors) and to their communities (e.g. reduced ecological harm and clean-up costs). Reducing litter generally, not only tobacco waste, may imbue communities with a sense of social order and safety^14^.

### Strengths and limitations

The present study provides new evidence of inequities in the exposure to tobacco waste among young people in a large, diverse, and recent sample. Among study limitations, while participants reported how often they noticed tobacco product litter, survey items did not ask them to specify the type of litter or where they saw it. It is possible that participants had different interpretations of the term ‘litter’. Also, online survey findings are not necessarily generalizable to all adolescents in California. For example, individuals particularly interested in the survey topic may have been more likely to participate (response bias). As an observational study, residual confounding cannot be ruled out.

## CONCLUSIONS

The study findings suggest that exposure to tobacco waste remains widespread, even in a state with a history of strong and effective tobacco control measures. As called for elsewhere, policy measures that take a top-down approach, such as banning the manufacture and sale of cigarette filters, may prove more effective at protecting environments than placing responsibility on individuals not to litter^3^. Given how often youth observe tobacco waste as suggested by this study, achieving a meaningful reduction in tobacco litter would offer a substantial public health impact.

## Data Availability

The data supporting this research are available from the authors on reasonable request.
